# Statistical Approaches to Detecting and Analyzing Tandem Repeats in Genomic Sequences

**DOI:** 10.3389/fbioe.2015.00031

**Published:** 2015-03-17

**Authors:** Maria Anisimova, Julija Pečerska, Elke Schaper

**Affiliations:** ^1^Institute of Applied Simulation, School of Life Sciences and Facility Management, Zürich University of Applied Sciences (ZHAW), Wädenswil, Switzerland; ^2^Department of Biosystems Science and Engineering, ETH Zürich, Basel, Switzerland; ^3^Department of Computer Science, ETH Zürich, Zürich, Switzerland; ^4^Vital-IT Competency Center, Swiss Institute for Bioinformatics, Lausanne, Switzerland

**Keywords:** tandem repeats, molecular evolution, protein domain, tandem repeat annotation, sequence profile model

## Abstract

Tandem repeats (TRs) are frequently observed in genomes across all domains of life. Evidence suggests that some TRs are crucial for proteins with fundamental biological functions and can be associated with virulence, resistance, and infectious/neurodegenerative diseases. Genome-scale systematic studies of TRs have the potential to unveil core mechanisms governing TR evolution and TR roles in shaping genomes. However, TR-related studies are often non-trivial due to heterogeneous and sometimes fast evolving TR regions. In this review, we discuss these intricacies and their consequences. We present our recent contributions to computational and statistical approaches for TR significance testing, sequence profile-based TR annotation, TR-aware sequence alignment, phylogenetic analyses of TR unit number and order, and TR benchmarks. Importantly, all these methods explicitly rely on the evolutionary definition of a tandem repeat as a sequence of adjacent repeat units stemming from a common ancestor. The discussed work has a focus on protein TRs, yet is generally applicable to nucleic acid TRs, sharing similar features.

## Tandem Repeats in Genomic Sequences

A tandem repeat (TR) in genomic sequence is a subsequent recurrence of a single sequence motif. TRs are described by the length of the minimal repeating motif (unit), the number of units, and the similarity among its units. The similarity of initially identical TR units fades with time through point mutations and indels, masking their shared ancestry. Diverged TR units, even when unrecognizable by eye, can maintain structural similarity over long evolutionary times [e.g., Figure 1 in Kajava (2012)]. While the mechanisms shaping TRs are poorly understood, they can evolve by duplication/loss of TR units, recombination, and gene conversion (Pearson et al., 2005; Richard et al., 2008). TRs can mutate by replication slippage (Levinson and Gutman, 1987; Ellegren, 2000), whereby the mispairing of a slipping-strand during the DNA synthesis causes a loss or gain of units as loops of TR units form hairpin structures (Mirkin, 2006).

**Figure 1:**
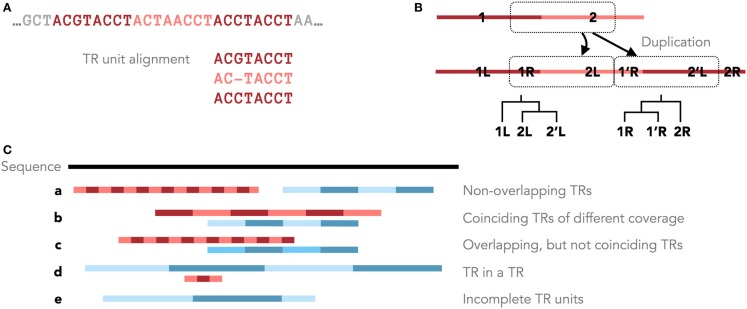
**Tandem repeats in genomic sequences. (A)** An example TR with three units and the corresponding MSA of its units. **(B)** Different parts of a TR motif (R = right and L = left) have different histories after a single duplication with shifted TR units. Shown are these duplication histories as phylogenies of the right and left parts of the TR motif. **(C)** Five scenarios of overlapping and non-overlapping TR annotations.

Tandem repeats are frequent in coding and non-coding DNA in species throughout the kingdoms of life. Genomic TRs are a rich source for genetic variability, providing a wide range of possible genotypes at a given locus (Nithiananthrajah and Hannan, 2007) and apt opportunity for selection, not only on long evolutionary scales but also during somatic cellular processes. Particularly staggering variation in TR unit lengths and numbers is characteristic to genomic TRs, such as ribosomal DNA arrays crucial for the translation machinery, and satellite DNA comprising the main component of functional centromeres (Richard et al., 2008). In protein-coding genes, mutations in TRs are likely to alter the structure/function of the protein product. Even in non-coding TRs, mutations can have serious fitness consequences by affecting gene regulation, transcription, and translation (Usdin, 2008). Crucially, TRs have attracted attention because of their medical relevance: many human proteins with TRs have been linked to monogenic disorders, typically affecting the nervous system (Siwach and Ganesh, 2008; Hannan, 2010). There is a high incidence of TRs in virulence factors of pathogenic agents, toxins, and allergens (Jorda et al., 2010).

## The Methodological Challenges for Tandem Repeats Detection

Systematic analyses of genomic TRs will help to better understand the biological processes governing and governed by TRs and their functional relevance. Such studies rely on the large-scale TRs detection (TRD). Numerous methods for TRD have been developed. Yet, since they are based on different algorithmic paradigms and heuristics, there is a large discrepancy between TR annotations produced by different algorithms for the same sequence (Leclercq et al., 2007; Merkel and Gemmell, 2008; Mudunuri et al., 2010; Schaper et al., 2012). For example, with four TRD methods applied to the human proteome, the majority of TRs were annotated by a single detector, only 9.8% were annotated by two and a meager 1.1% by at least three (Schaper et al., 2012).

The TR heterogeneity contributes to the large variability among TRD methods. The TRD is relatively simple for identical units. If the TR motif is unknown, this task is computationally expensive for long sequences, requiring an exhaustive search with no information on TR unit length, number, or position in the sequence (search space in O(*N*^3^) for sequence length *N*). Substitutions and indels in the TR region cause major challenges to TRD: with decreasing unit similarity, TR regions become hard to discern. Indels introduce length variability between individual TR units, increasing the TR search space to O(2*^N^N*^3^).

Furthermore, the original TR unit boundaries can be shifted due to new unit duplications (Figure 1A, Benson and Dong, 1999; Rivals, 2004). Clear boundaries are preserved only in some cases, for example, when protein TRs are confined by the exon structure. Therefore, unambiguously dividing a TR region into units of similar lengths may not accurately reflect the TR duplication history. Occasionally, the TR history is described by different phylogenies for different parts of the TR motif (Figure 1B). Thus, defining the consensus TR motif is problematic, and TRD methods typically differ in the predicted unit lengths and boundaries.

Ultimately, TRD methods differ by TR definitions. One TR definition borrows from string matching in computer science, whereby TRs are defined by a repetitive regular expression, allowing for a fixed proportion of dissimilar characters among TR units. This viewpoint enables straightforward exhaustive TRD algorithms, but lacks biological interpretation. Alternatively, from a structure perspective, protein TRs may be defined by structural repetitions, which allows TR detection for structurally conserved TR units, even with low sequence similarity [see, e.g., the structural TR database Repeats DB, Di Domenico et al. (2014)]. Yet, defining structural repeats is in itself problematic. Finally, the evolutionary definition states that a TR stems from ancestral unit duplications. This viewpoint has a direct biological interpretation reflecting the TR generating mechanism. Most TRD methods, however, lack an explicit TR definition, which obscures the search objective and TRs are typically detected by empirical properties of unit similarity. This further impedes the ability to evaluate the statistical properties of a method. Improving TRD requires a rigorous statistical framework based on a clear TR definition described as a biologically meaningful mathematical model. Then, a genuine TR can be distinguished from a non-TR sequence by comparison with a model describing random sequences. Relying on a mathematical TR model means that the TRD method’s behavior can be predicted for different scenarios, including the evaluation of false predictions.

One possibility is to define TR units as related by a common ancestral unit under a Markov substitution model and a standard phylogeny model reflecting the unit duplication history (Schaper et al., 2012). The evolutionary distance *t* from currently observed TR units to their common ancestor can be estimated by maximum likelihood. For any tentative TR region with predicted units, this conveniently allows for statistical hypothesis testing using a likelihood ratio test (LRT; Schaper et al., 2012). The null hypothesis “*t* = ∞” represents that the estimated evolutionary distance is so large that TR units have no common origin and could have appeared by chance. Rejecting the null suggests that the given units are related (with finite *t*) and therefore are assumed to be TRs by definition. Such approach provides a statistical framework to validate predicted TRs, filtering out potential false positive predictions.

The variability in TR annotations produced by different TRD methods warns against relying on one specific algorithm. Different methods not only achieve optimal power for different combinations of TR divergence and unit length, but also vary in their accuracy across the TR space. Therefore, to obtain the most complete and accurate set of TR annotations for a given sequence, we suggest that multiple TR detectors should be used [maximizing the number of true positives, e.g., as in Pellegrini et al. (2012)] followed by validation with an LRT (controlling false positives at a fixed significance level).

## Annotating TRs with Sequence Profile Models

Many common protein domains found in tandem are listed in sequence profile databases, such as Pfam (Punta et al., 2011), PROSITE (Sigrist et al., 2010), SMART (Letunic et al., 2012), Repbase (Jurka et al., 2005), and Dfam (Travis et al., 2013). For example, of all *de novo* annotated TRs with unit length ≥15, we found that only few had not been described in Pfam (2.1% in *Arabidopsis thaliana* and 11.5% in human). Thus, TR annotation can profit strongly from the existing databases.

Profile-based annotation typically relies on sequence profile hidden Markov models (HMMs). Circular connections in an HMM allow the annotation of full TR units (Bucher et al., 1996; Schaper et al., 2014), as implemented in pftools (Sigrist et al., 2013) and in our Python TR Annotation Library TRAL (http://elkeschaper.github.io/tral/). General profile HMM annotation can be used to detect TR units [e.g., HMMER; Eddy (2011)], but a subsequent analysis is required to annotate the whole TR region, potentially including diverged TR units that, without considering the whole TR region, lack statistical significance.

Importantly, sequence profile HMMs can be used to refine *de novo* annotations. *De novo* detected TR units provide seed motifs that can be converted to sequence profile models (e.g., with *hmmbuild* from the HMMER package). These models can then be used to re-annotate sequences. The advantage is twofold: first, the quality of annotation is homogenized among TRs from different TRD methods; second, annotations on homolog sequences become comparable.

## An Example Pipeline for Meta-Prediction of Genomic TRs

**Figure 2:**
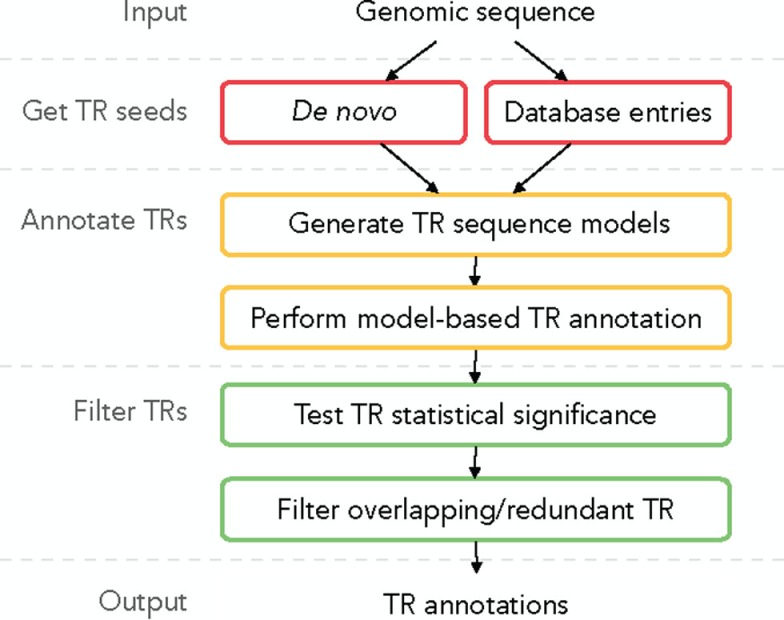
**Overview of a generic TR annotation workflow**.

A plausible TR annotation pipeline in three steps could be (1) identify a putative TR unit seed motif; (2) detect all tandem occurrences of this motif in the sequence, forming a putative TR; and (3) for each putative TR validate its statistical significance and filter out redundant predictions. We describe this TR annotation workflow in Figure 2; all functionalities are implemented in TRAL (http://elkeschaper.github.io/tral/).

Tandem repeat seed motifs are obtained from sequence profile databases and multiple *de novo* TRD algorithms. Circular profile HMMs are built from these TR seeds and consequently used to annotate TR regions in a sequence. All annotated TRs must be statistically validated, for example, using an LRT. A multiple testing correction may be required dependent on the application [e.g., Saville (1990)]. TRs that fail the test are assumed to be false positive predictions and are discarded. Combining several methods leads to redundant predictions, which is not limited to the rare case where two TR predictions fully coincide. Due to differences in predicted unit boundaries or unit numbers, it is often difficult to decide whether two overlapping TR annotations describe the same TR or, rather, nested or neighboring TRs (Figure 1C). To filter redundant predictions, several *ad hoc* criteria may be used. For example, overlapping TRs may be seen as redundant (the required degree of overlap demands another *ad hoc* decision; Figure 1C, b–d). Using the evolutionary TR definition, we propose one possible criterion based on the representation of a TR region as a multiple sequence alignment (MSA) of its TR units. Characters grouped in one MSA column are assumed to derive from a common ancestral character. Given this, one of two TR annotations may be seen as redundant if the characters in two annotations are grouped into columns similarly by the two corresponding MSAs (Figure 1C, b). Model-based test statistics may be defined to recognize redundant TRs, deciding if two independent TR models provide a better description of these TRs compared to one common model.

We used *de novo* and profile-based methods to annotate TRs in the entire UniprotKB/Swiss-Prot (UniProt Consortium, 2014, v2013-08) with the proposed pipeline (Figure 2). A considerable proportion of these sequences contain TRs: 46% in Eukaryotes, 29% in Archaea, and 27% in Bacteria. These TRs, across all kingdoms of life, are dominated by microsatellite (typically < 10 bp) or short minisatellite TRs (~10–100 bp) with few units.

## Probabilistic MSA with TRs

When aligning homologous sequences, often we are not aware of the presence of TRs. Yet, due to uneven TR unit gain/loss among the homologs, TR-containing MSAs are error-prone, since standard indel penalty schemes do not account for the potential variation in TR region length. Some MSA methods accounting for sequence repeats produce local alignments (Raphael, 2004; Phuong et al., 2006; Treangen et al., 2009). However, a global alignment is required for evolutionary inferences, such as for the estimation of TR unit history. Sammeth and Heringa (2006) proposed a global MSA method with fixed TR unit boundaries. Yet, unit boundaries may be distorted by indels, slippage, and recombination. Modeling TRs explicitly in the MSA graph representation allows TR units to start at any position, adequately penalizing indels corresponding to unit gains/losses, and to reconstruct the evolutionary history of TR unit events. The implementation ProGraphMSA + TR (Szalkowski and Anisimova, 2013) uses a probabilistic phylogeny-aware approach similar to PRANK (Löytynoja and Goldman, 2005), achieving not only improved alignment quality, but also *a posteriori* estimation of rates of evolutionary events, such as TR unit indels. For example, ProGraphMSA + TR was applied to leucine-rich repeats (LRRs) in a gene family of type III effectors determining the pathogenicity in agriculturally important bacteria *Ralstonia solanacearum*. The estimates of TR indel frequencies in different clades of a gene phylogeny suggested that TR indel rate variation contributes to the diversification of this protein family [Figure 9 of Szalkowski and Anisimova (2013)]. Variation in LRR unit numbers might contribute to adaptive processes in this gene and to pathogenesis on different plant hosts.

## Phylogenetic Approach to Study the Evolution of TRs

Tandem repeat unit phylogenies reconstructed from homologous TRs carry much evolutionary signal, even with short units (Schaper and Anisimova, 2014; Schaper et al., 2014). These phylogenies inform about unit duplication histories and TR unit gain/loss rates, allowing to study selection on TRs and their functional relevance. Clustering patterns in TR unit phylogenies describe the unit conservation between species. If the TR unit number and order in orthologs regions from different species are perfectly conserved throughout the evolution, then the phylogeny of all TR units consists of clades formed by orthologous unit copies, each reflecting the phylogeny of the whole region (or species) [Figure 1B of Schaper et al. (2014)]. In contrast, if TR regions are fully separated, then a speciation event is followed by a series of TR unit gain/loss events, and the TR unit phylogeny consists of species-specific monophyletic clades of TR units [Figure 1A of Schaper et al. (2014)].

Tandem repeat unit phylogenies from pairs of orthologs can be used to backtrack the evolution of TRs from a single species (Schaper et al., 2014) or across an entire species tree – using the all-against-all pair-wise approach (Schaper and Anisimova, 2014). In contrast to multispecies TR unit phylogenies, for pair-wise TR unit phylogenies, the statistical significance of observing perfect conservation and separation patterns is computed exactly (Schaper et al., 2014). On the other hand, multispecies TR unit phylogenies suffer fewer reconstruction errors due to the additional information on the unit evolution from additional orthologs.

Our large-scale analyses of eukaryotic proteomes revealed an extremely deep conservation of some protein domain TRs (dTRs), many dating to hundreds million years ago and some even to the times of separation between human and yeast (0.6–1.6 billion years ago) or red algae and green plants (~1.6 billion years ago). Conserved dTRs span much of the TR diversity of proteomes. For example, in human 81% of detected distinct dTR types have been conserved at least to the ancestor of mammals at least in one protein (Schaper et al., 2014). The distribution of conserved domain types is highly heterogeneous: 68% of all conserved dTRs are described by only 5% of all TR types detected in human. Yet, many conserved TRs are rare and occur only in a single protein. Similar numbers were observed in plants (Schaper and Anisimova, 2014).

In contrast, very few dTRs have separated between closely related species. In human, dTRs separated within mammals are dominated by zinc finger repeats (~50%), followed by DUF1220 (~8%; Schaper et al., 2014). In *A. thaliana*, dTRs separated within magnoliophytes are dominated by LRRs (~40%), followed by ankyrin (~12%) (Schaper and Anisimova, 2014). For both species, separated dTRs are enriched in *de novo* annotations, i.e., rare and presumably recent dTRs, which are perhaps more prone to unit gains/losses due to relaxed selection or due to higher mutation rates as a result of a high among-unit sequence similarity.

## Simulating Genomic TRs

Benchmarking and hypothesis testing in bioinformatics must often rely on simulated data since the truth is rarely known. For example, only the observation of identical TR units qualifies them as a true TR with certainty. Yet this is only the simplest scenario, which is of little practical relevance. With diverged or shifted TR units, unbiased benchmarks are challenging to construct from real data. Model-based simulation offers a powerful means to benchmarking and hypothesis testing in bioinformatics studies of TRs. Simulations enable comparisons of competing hypotheses and help to reveal methodological weaknesses or detect and estimate important factors. Simulations are not only crucial for benchmarking new TRD methods, but also to study the underlying evolutionary mechanisms of genomic TRs. For example, to describe the biological mechanisms for specific TR types, patterns or parameter estimates observed in these data can be compared with those obtained from sequences simulated with alternative models of TR evolution. Sequences with TRs may be generated not only with the dedicated models of evolution by unit gain/loss with fuzzy units boundaries, e.g., SlippageSim (Szalkowski and Anisimova, 2013), but also with other general sequence simulators that allow gene family evolution by mutations, indels, gene gain/loss, recombination, etc. [e.g., Dalquen et al. (2012)]. For example, sequences with TRs of different divergence and unit lengths were used to benchmark the MSA method ProGraphMSA + TR that accounts for sequence TRs (see above). The evaluation of the power of TRD methods also relies on simulated sequences with TRs (the alternative hypothesis). Yet the high power is irrelevant without the evaluation of false positive TRD rates, which must be done on TR-free data (the null hypothesis). Furthermore, simulated TR-free data helps to validate TR-specific findings: the comparison of patterns found in simulated TR-free sequences with those observed in sequences with TRs serves to disentangle TR-specific findings from those that may occur in genomic sequences in general. A simple approach is to simulate TR-free data by drawing *k*-mers from a (*k* - 1)-th order Markov model based on empirical frequencies (Robin et al., 2007). In contrast to drawing single characters from their frequency distribution, simulating *k*-mers mimics natural local correlations while choosing small *k* minimizes the chance of hidden TRs within a *k*-mer.

## Conclusion and Perspectives

Tandem repeats are diverse in their size, type, unit similarity, and distribution across genomes. Methods discussed above enable accurate detection of TR orthologs with strongly conserved unit configurations, or on the contrary, with highly changing unit numbers. Due to the heterogeneity of TRs, large-scale studies should be followed up by studies that focus on specific TR types and their effects on molecular processes. Our TR scans of eukaryotic proteomes provide a plentitude of cases to investigate with respect to their functional roles. While many TRs were linked to key functions, phenotypic changes, or disease predisposition, the biological mechanisms generating and preserving TRs in genomes are poorly understood. New studies of genomic TRs only fuel our fascination with these genomic features, calling for further research and for the development of dedicated methods. Further development of rigorous statistical models of TR generating mechanisms will help to improve TRD methods, and to shed some light on biological forces shaping these sequences.

## Conflict of Interest Statement

The authors declare that the research was conducted in the absence of any commercial or financial relationships that could be construed as a potential conflict of interest.
